# Autonomic correlates of seeing one’s own face in patients with disorders of consciousness

**DOI:** 10.1093/nc/niv005

**Published:** 2015-07-30

**Authors:** Sergio Bagnato, Cristina Boccagni, Caterina Prestandrea, Giuseppe Galardi

**Affiliations:** Unit of Neurophysiology and Unit for Severe Acquired Brain Injuries, Rehabilitation Department, Fondazione Istituto “San Raffaele - G. Giglio,” Viale G. Giardina, 90015 Cefalù (PA), Italy.

**Keywords:** autonomic nervous system, minimally conscious state, sympathetic skin response, unresponsive wakefulness syndrome, vegetative state.

## Abstract

The ability to recognize one’s own face is a hallmark of self-awareness. In healthy subjects, the sympathetic skin response evoked by self-face recognition has a greater area under the curve of the signal than responses evoked by other visual stimuli. We evaluated the sympathetic skin responses evoked by self-face images and by six other visual stimuli (conditions) in 15 patients with severe disorders of consciousness and in 15 age-matched healthy subjects. Under all conditions, the evoked area of the sympathetic skin response was smaller in patients with unresponsive wakefulness syndrome, intermediate in patients in a minimally conscious state, and greater in healthy subjects. In patients with unresponsive wakefulness syndrome, no differences were found between the sympathetic skin response area evoked by self-face images and those evoked by other conditions. In patients in a minimally conscious state, the area of the sympathetic skin response evoked by self-face presentation was greater than those evoked by other conditions, even if statistical significance was reached only in the comparison to other stimuli not involving a real face. This finding may be due to the inability of these patients to differentiate their own face from those of others. Taken together, these results probably reflect a varying level of self-awareness between patients with unresponsive wakefulness syndrome and patients in a minimally conscious state, and suggest that the autonomic correlate of self-awareness may have some diagnostic implications for these patients.

## Introduction

Distinguishing patients with a very low level of consciousness (i.e. who are in a minimally conscious state [MCS]) from patients who are fully unconscious (i.e. who have unresponsive wakefulness syndrome [UWS]) can be a difficult task. UWS (previously known as vegetative state) and MCS can result from severe brain injuries that deeply affect the ability of the brain to generate consciousness. Patients with UWS are able to open their eyes spontaneously, but they lack any sign of self- or environmental awareness (Royal College of Physicians, 2003). The MCS represents the lowest level of consciousness in which awareness can be detected. Patients in an MCS display inconsistent but clear evidence of awareness, such as ocular fixation, localization of noxious stimuli, intelligible verbalization, intentional communication, or the ability to follow simple commands (Giacino *et al.*, 2002).

Differential diagnosis between UWS and MCS raises serious problems, because the current diagnostic criteria are still based on a careful (but subjective) clinical assessment of the patient’s spontaneous and elicited behaviors (Giacino *et al.*, 2002; Royal College of Physicians, 2003). Diagnostic errors arise from the difficulty of assessing low levels of responsiveness because conscious behavior may be highly variable, especially in the first phases of emersion from UWS, or because motor (e.g. paralysis) or cognitive deficits (e.g. aphasia) may prevent the patient from demonstrating consciousness in specific assessment tasks (Giacino *et al.*, 2013). As a result, an alarmingly high rate of misdiagnosis with UWS has been reported in patients who were actually in an MCS (Schnakers *et al.*, 2009).

A way to discover some potential signs of awareness in noncommunicative patients is to gain access to their emotional contents by evaluating their autonomic responses, such as through the sympathetic skin response (SSR) evoked by a visual stimulus. The SSR is generated in the deep layers of the skin by a reflex activation of the sweat glands via cholinergic sudomotor sympathetic efferents (Vetrugno *et al.*, 2003). Although the central organization of the SSR is not completely understood, the SSR is likely to be influenced by inputs from the basal ganglia, the premotor cortex, the temporal and frontal cortexes, as well as the hypothalamus and limbic system (Claus and Schodorf, 1999; Vetrugno *et al.*, 2003). The SSR is easily recorded via electrodes placed on a hand or foot, and SSR recording is considered to be a noninvasive approach for investigating the function of the sympathetic system. For clinical purposes, the most commonly used stimulus to evoke the SSR is an electric shock delivered to a peripheral nerve, most frequently the median or ulnar nerve. However, several other modalities may evoke an SSR, such as mental calculation, deep breathing, an acoustic stimulus or flash, and other forms of somatic or mental stress (Vetrugno *et al.*, 2003). As the SSR may be deeply affected by the subject’s emotional state, it is usually considered a psychophysiological response.

Recently, we elaborated a new paradigm for evaluating the sizes (areas) of SSRs evoked by the recognition of one’s own face, or “self-face,” compared to other faces or images (conditions) (Bagnato *et al.*, 2010). The most interesting result was that the SSR size was greatest when participants were shown their face compared to other stimuli. This result was probably due to a peculiar interaction between the cerebral areas involved in self-face recognition and the structures of the sympathetic vegetative system implicated in the genesis of SSRs (Bagnato *et al.*, 2010). In this study, we tested the hypothesis that SSRs evoked by different visual stimuli, including one’s own face, may be useful in differentiating conscious from unconscious behaviors in patients with severe disorders of consciousness.

## Methods

### Participants

As candidates for this study, we evaluated 21 consecutive patients with severe disorders of consciousness who were admitted to our Unit for Severe Acquired Brain Injuries. All patients who fulfilled the following criteria were included in the study: (i) diagnosis of UWS or MCS by the revised Coma Recovery Scale after an acute brain injury (Giacino *et al.*, 2004); (ii) age under 50 years (the SSR area may be reduced in older subjects); (iii) presence of an SSR evoked by an electric shock; and (iv) normal visual evoked potentials obtained by flash stimulation and sensory nerve conduction studies in the upper limbs (i.e. sensory nerve action potential of the ulnar nerve). Patients with a previous history of vision impairment or neuropathy were not evaluated as candidates for this study. Four patients with abnormal nerve conduction studies, 1 patient with abnormal nerve conduction studies and absent SSR evoked by electric shock, and 1 patient with abnormal visual evoked potentials were excluded from the study. Hence, 7 patients with UWS and 8 patients in an MCS (4 females and 11 males, mean age 32 ± 6 years) participated in the study (see Table 1 for a detailed description). Data obtained from patients were compared with those acquired from 15 age-matched healthy subjects (7 females and 8 males, mean age 31.3 ± 3.6 years).

**Table 1:** patients demographic and clinical characteristics

Patient	Gender	Age (years)	Etiology	Time from brain injury (in months)	Disorder of consciousness	CRS-*R* score	Lesions (CT and/or MRI findings)
1	F	20	TBI	8	UWS	4	Diffuse axonal injury; multiple intraparenchymal microhemorrhages.
2	M	36	TBI	7	UWS	3	Right temporo-parietal intraparenchymal hemorrhage; multiple intraparenchymal microhemorrhages; brainstem hemorrhage.
3	M	35	TBI	10	UWS	3	Diffuse axonal injury; multiple intraparenchymal microhemorrhages.
4	F	31	Cerebral hypoxia	15	UWS	4	Cerebral edema.
5	M	33	TBI	7	UWS	4	Diffuse axonal injury; multiple intraparenchymal microhemorrhages.
6	M	27	TBI	2	UWS	5	Bilateral frontal epidural hematoma; bilateral frontal cortical contusions; right frontal intraparenchymal hemorrhage.
7	M	33	TBI	2	UWS	5	Multiple intraparenchymal microhemorrhages; right frontal subarachnoid hemorrhage.
8	M	29	TBI	13	MCS	10	Right hemispheric epidural hematoma; left parietal intraparenchymal hemorrhage.
9	F	22	TBI	39	MCS	14	Diffuse axonal injury; multiple intraparenchymal microhemorrhages.
10	M	22	TBI	12	MCS	13	Diffuse axonal injury; multiple intraparenchymal microhemorrhages.
11	M	17	TBI	8	MCS	14	Diffuse axonal injury; multiple intraparenchymal microhemorrhages.
12	M	29	TBI	19	MCS	14	Left fronto-temporal epidural hematoma.
13	M	21	TBI	9	MCS	12	Right fronto-temporal subdural hematoma.
14	M	25	TBI	5	MCS	20	Multiple cerebral contusions.
15	F	32	AVM rupture	3	MCS	17	Left frontal subarachnoid hemorrhage; hydrocephalus.

AVM, arteriovenous malformation; CRS-R, Coma Recovery Scale Revised; MCS, minimally conscious state; TBI, traumatic brain injury; UWS, unresponsive wakefulness syndrome.

This study was approved by the ethics committee of Fondazione Istituto “San Raffaele - G. Giglio” (Cefalù, Italy). Healthy subjects and the patients’ legal guardians gave their written informed consent to all procedures.

### Experimental procedures

Details of the experimental protocol are reported elsewhere (Bagnato *et al.*, 2010). In brief, a digital picture of the face was obtained for each participant (in patients, pictures were from before the brain injury). During the experiment, participants were comfortably seated on a chair and connected to an electromyograph for recording of SSRs induced by visual stimuli. Participants were seated at a distance of ∼3 meters from, and facing, a white wall on which a video projector (total screen area about 112 × 84 cm) projected images.

Seven conditions were previously defined, each consisting of the presentation of a different image: (i) a board made up of 900 alternating black and white rectangles; (ii) a photo of an attacking snake; (iii) the stylized face of a man drawn in black pencil on a white background; (iv) a reproduction of Munch’s picture “The Scream”; (v) the smiling face of an unknown person of the same gender as the participant; (vi) the face of a famous actor/actress of the opposite gender to the participant (Brad Pitt or Angelina Jolie); and (vii) a photo of the participant’s own face (Bagnato *et al.*, 2010). Condition 1 (“board”) was chosen to study the subject’s reaction during a neutral visual stimulus, without emotional content. Condition 2 (“snake”) was selected to evoke a feeling of fear. Condition 3 (“stylized face”) was included to study the response to the presentation of an archetypal face. Condition 4 (“scream”) was used to allow investigation of two major aspects: (i) recognition of a famous work of art; and (ii) identification of a face on a background painted in similar colors. Condition 5 (“unknown”) was intended to study the subject’s response to the face of a stranger of his/her own gender. Condition 6 (“well-known”) was selected to analyze the subject’s response to the face of a well-known actor/actress of the opposite gender. Finally, condition 7 (“self-face”) was used to evaluate the participant’s response to seeing a picture of his/her own face.

**Figure 1:**
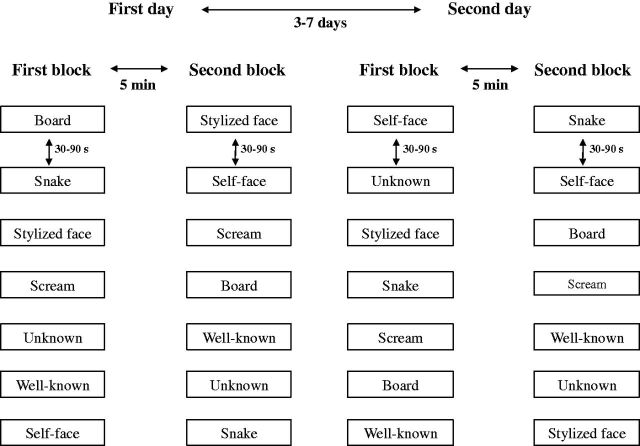
Schematic representation of the study design Different visual stimuli were delivered in two sessions separated by an interval of 4–7 days. Each session consisted of two blocks organized to value the SSRs induced by all seven conditions (“board,” “snake,” “stylized face,” “scream,” “unknown,” “well-known,” and “self-face”). The time between two conditions was 30 and 90 seconds in pseudorandom order. The time interval between two blocks was 5 min. In each block, the presentation order of the conditions was randomized. Adapted with permission from Bagnato *et al.,* 2010.

The experiment consisted of two recording sessions performed on different days. Each session was comprised of two blocks designed to evaluate the SSRs induced by each of the seven conditions described above. During each experimental session, a condition was projected onto a white wall in front of the participants for 5 seconds, followed by a black slide. SSR recording began the moment each image appeared. The interval between two different conditions was set between 30 and 90 seconds in a predetermined pseudorandom order, but supervised by the investigator. If spontaneous SSRs were observed (e.g. due to deep inspiration), the visual stimulus was not delivered. A break of 5 minutes was allowed between the first and second blocks, during which the subject was invited to relax. After 3–7 days, the protocol was repeated in a second session, with the slides being shown again in a different random order. Figure 1 shows a schematic representation of the experimental design.

### Data acquisition and analysis

The SSRs evoked by the visual stimuli were recorded with a standard electromyograph (Micromed System PLUS Evolution, Mogliano Veneto, TV, Italy). The SSRs were acquired bilaterally (bandpass 0.1–10 Hz) from a pair of Ag–AgCl surface electrodes placed on the participant’s hand, with the cathode on the center of the palm and the anode on the corresponding region on the back of the hand. The ground electrode was placed on the right forearm. For each recorded SSR, the area under the curve of the signal (measured for all peaks and expressed in microvolts per second) was analyzed.

Single subjects data are reported as the mean of the SSRs area recorded from both hands in all sections (four recording blocks) ± SD. The purpose of the statistical analysis was to evaluate whether the characteristics of SSRs induced by different visual stimuli were related to the conditions (the seven projected images) and to the different groups (healthy subjects, patients in an MCS, patients with UWS). Thus, a repeated-measures analysis of variance (ANOVA) with the factor “condition” (seven levels: condition 1 vs. condition 2, vs. condition 3,… vs. condition 7) was used as the within-subjects factor and the factor “group” (three levels: healthy subjects vs. patients in an MCS, vs. patients with UWS) as the between-subjects factor. For each condition, the mean of the SSRs recorded from both hands in all sections (four recording blocks) was considered in the statistical analysis. Mauchly’s test was used to evaluate the sphericity of variances. The Greenhouse–Geisser method was applied, if necessary, to correct for nonsphericity. Conditional on a significant *F*-value, *posthoc* comparisons were performed with the Tukey honest significant difference test. For all analyses, a *P* < 0.05 was considered statistically significant.

### Data availability statement

Data is available on request.

## Results

The SSR area evoked by one’s own face was greater than those evoked by all other conditions in 14 of the 15 healthy subjects (93.3%), 5 of the 8 MCS patients (62.5%), and 1 of the 7 UWS patients (14.3%). The three conditions involving a real face (i.e. condition numbers 5, 6, and 7) included the greatest SSR area in 7 of the 8 MCS patients (87.5%; random distribution = 37.5%) and in 4 of 7 UWS patients (57.1%; random distribution = 42.9%) (Table 2).

**Table 2:** area of the SSRs evoked by different visual stimuli (conditions) in patients with UWS or in an MCS.

Patient	Cond 1	Cond 2	Cond 3	Cond 4	Cond 5	Cond 6	Cond 7
Patients with UWS							
1	28 ± 32	13 ± 27	**520 ± 1041**	71 ± 58	78 ± 59	41 ± 35	8 ± 17
2	80 ± 84	31 ± 62	103 ± 89	49 ± 98	63 ± 106	**599 ± 748**	160 ± 122
3	29 ± 39	25 ± 50	25 ± 50	0	15 ± 22	**52 ± 66**	4 ± 8
4	48 ± 56	96 ± 71	32 ± 65	16 ± 33	**709 ± 1227**	543 ± 655	182 ± 233
5	145 ± 204	0	0	**593 ± 179**	0	0	0
6	0	0	58 ± 82	14 ± 19	100 ± 141	0	**148 ± 229**
7	29 ± 21	47 ± 16	41 ± 11	**48 ± 39**	36 ± 16	39 ± 23	33 ± 10
Patients in an MCS							
1	912 ± 1071	157 ± 108	250 ± 335	98 ± 49	582 ± 967	1237 ± 835	**3054 ± 2160**
2	0	21 ± 30	136 ± 192	92 ± 26	75 ± 16	59 ± 45	**152 ± 48**
3	43 ± 60	768 ± 1085	748 ± 1058	104 ± 147	**2558 ± 93**	776 ± 181	1326 ± 586
4	1041 ± 688	1044 ± 1266	711 ± 683	634 ± 407	1703 ± 1208	729 ± 360	**4220 ± 5926**
5	42 ± 51	52 ± 60	69 ± 117	12 ± 23	**90 ± 85**	31 ± 48	20 ± 39
6	0	0	0	0	0	0	**28 ± 39**
7	7 ± 15	13 ± 15	45 ± 13	23 ± 26	32 ± 50	29 ± 22	**48 ± 27**
8	133 ± 72	98 ± 76	130 ± 123	**216 ± 260**	145 ± 134	76 ± 30	126 ± 85

Cond 1 = “board”; Cond 2 = “snake”; Cond 3 = “stylized face”; Cond 4 = “scream”; Cond 5 = “unknown”; Cond 6 = “well-known”; Cond 7 = “self-face”. For each condition, the mean of the SSRs area recorded from both hands in all blocks is reported (± SD). For each patient, the greater value of the SSR area among the different conditions is indicated in bold. Area is given in microvolts per second. MCS, minimally conscious state; UWS, unresponsive wakefulness syndrome.

**Figure 2:**
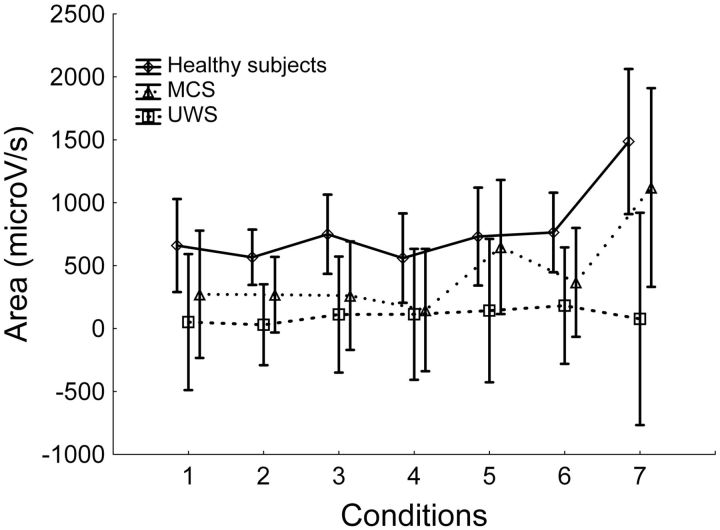
Area amplitude of the SSRs evoked by different visual stimuli (conditions) in healthy subjects and patients For each condition, the mean values resulting from both hands in all blocks (eight SSRs per condition) are reported. Vertical lines denote 95% confidence intervals. Condition 1 = “board,” condition 2 = “snake,” condition 3 = “stylized face,” condition 4 = “scream,” condition 5 = “unknown,” condition 6 = “well-known,” and condition 7 = “self-face,”. MCS, minimally conscious state; UWS, unresponsive wakefulness syndrome.

ANOVA showed a significant effect for the two main factors. The significant effect of the factor “condition” (*F*_6,162_ = 6.3; *P* < 0.001) was attributable to the larger SSR area evoked by seeing the self-face in healthy subjects and in MCS patients (Fig. 2). *Posthoc* analysis demonstrated that the SSR area evoked by the self-face was significantly greater than that evoked by the other conditions (“face” vs. “board,” “snake,” “stylized face,” “scream,” “unknown,” or “well-known,” *P* < 0.001). No significant effects were found between the other conditions.

The significant effect of the factor “group” (*F*_2,27_ = 3.5; *P* = 0.04) was due to the overall SSR area, which was greater in healthy subjects, intermediate in MCS patients, and smaller in UWS patients (Fig. 1). *Posthoc* analysis showed a significant difference between healthy subjects and patients with UWS (*P* = 0.04), but no significant differences between healthy subjects and MCS patients (*P* = 0.4) or between MCS patients and UWS patients (*P* = 0.5).

No significant “condition-by-group” interaction was found (*F*_12,162_ = 1.6; *P* = 0.08). This result was attributable to the fact that both healthy subjects and MCS patients had greater SSR areas evoked by presentation of the self-face compared to the other conditions (Fig. 2). Although patients with UWS showed a different result (i.e. no difference among SSR areas evoked by self-face compared to the other conditions), this result was not sufficient to achieve a significant “condition-by-group” interaction among the three groups.

*Post* *hoc* analysis demonstrated that the SSR area evoked by self-face presentation was always significantly greater than that evoked by the other conditions in healthy subjects (“self-face” vs. “board,” “snake,” “stylized face,” “scream,” “unknown,” or “well-known,” *P* < = 0.01). In MCS patients, the SSR area evoked by self-face presentation was significantly greater than that evoked by the other conditions only when recognition of a real face was not required (i.e. “self-face” vs. “board,” “snake,” or “stylized face,” *P* = 0.02; “face” vs. “scream,” *P* < 0.01). Conversely, no significant differences were found between the SSR areas evoked by presentation of the self-face and presentation of a real face (i.e. “self-face” vs. “unknown” *P* = 0.9; “face” vs. “well-known” *P* = 0.09). No significant differences were found between the SSR areas evoked by presentation of the self-face compared to other conditions in patients with UWS. All *posthoc* analyses are reported in the Supplementary data.

## Discussion

In this study, we found that the SSRs evoked by visual stimuli clearly differed, both quantitatively and qualitatively, between fully unconscious and fully conscious subjects (i.e. UWS patients and healthy subjects). Indeed, (i) the total SSR area was smaller in UWS patients than in healthy controls, and (ii) UWS patients, unlike healthy subjects, did not have a greater SSR area compared to the other conditions when their own face was shown. Patients in an MCS exhibited an intermediate behavior; the SSR area evoked by self-face presentation was greater than those evoked by other conditions, but this difference was statistically significant only when compared to conditions not involving the presentation of a real face.

In UWS patients, the highly reduced size of the SSRs reflects the disruption of the body–mind interactions due to a lack of external world awareness. It is likely that the extremely low-SSR areas obtained in response to visual stimuli denote the effect of the baseline electrodermal activity only, without any correlation with the presented stimuli. In healthy subjects, the brain areas involved in cognitive and affective processes are strictly coupled with the cortical and subcortical regions that mediate autonomic responses, such as the insular and cingulate cortices, the amygdala, and the dorsal pons (Critchley *et al.*, 2013). However, UWS patients are characterized by multilevel brain disconnection. In particular, several recent electroencephalographic and neuroimaging studies have suggested that UWS involves impaired operational synchrony among neuronal assemblies in multiple brain areas (Sarà *et al*., 2011; Fernández-Espejo *et al.*, 2012; Fingelkurts *et al.*, 2012a,b; Bagnato *et al.*, 2013; Demertzi *et al.*, 2014). In this context, the unawareness of external stimuli parallels the lack of further integrative processes and the impossibility of enriching these stimuli with autonomic correlates. Moreover, this result did not change when one’s one face was shown, a condition that induced greater SSR areas in healthy subjects and MCS patients.

Patients in an MCS exhibited greater SSR areas than would be expected from simple baseline electrodermal activity. Like the healthy subjects, MCS patients displayed the largest SSR area when their own face was shown. This effect was statistically significant compared with the other conditions without a real face. Although the SSR area was also greater than the other conditions involving a real face, statistical significance was not reached. These results probably reflect the human brain characteristic of having different neural circuits for the elaboration of faces compared to other visual stimuli.

A critical point regards whether these different responses truly reflect a different level of self-awareness in patients in an MCS. Previous studies suggested that specific neurophysiological responses to stimulations with an emotional content may be induced during sleep or in unconscious patients (Perrin *et al.*, 1999; Kotchoubey *et al.*, 2009). Although we cannot provide an unquestionable refutation to the hypothesis of an unconscious processing of faces in MCS patients, some data from the present study and from the literature should be considered. In particular, we found different patterns of SSR responses between fully unconscious patients (i.e. patients with UWS) and minimally conscious patients (i.e. patients in an MCS) when we compared the SSR evoked by the self-face with the SSR evoked by conditions not involving faces. This result suggests that the greater SSRs evoked by the self-face in MCS patients is related directly (i.e. a conscious processing of faces occurs), or at least indirectly (i.e. a conscious processing of faces does not occur, but the presence of a minimal level of consciousness is required), to a different level of awareness. The capability of cortically processing salient stimuli, such as one’s own name or face, is not unequivocally associated with the presence of awareness, because it has been described in UWS patients (Perrin *et al.*, 2006; Sharon *et al.*, 2013; Wang, 2015). However, this cortical processing probably characterizes a subpopulation of patients who have better chances of recovering consciousness (Menon *et al.*, 1998; Sharon *et al.*, 2013; Wang, 2015). Interestingly, it was recently proposed that the brain areas engaged in face processing are also involved in several other high-level cognitive functions (Kanwisher, 2010), which are unlikely to be present in fully unconscious subjects. As a consequence, the use of the self-face as a stimulus is considered particularly suitable for self-awareness evaluation in patients with disorders of consciousness (Laureys *et al.*, 2007).

For better clarification of how SSRs during face presentation correlate with the level of consciousness, future studies might evaluate the profiles of responses in MCS patients with different degrees of awareness impairment. Indeed, it was recently proposed that MCS patients may be differentiated into categories of “MCS plus” (patients who exhibit a higher level of purposeful behavior) and “MCS minus” (patients who exhibit a lower level of purposeful behavior) (Bruno *et al.*, 2011). This categorization reflects a different level of awareness, probably related to a different pattern of neuroanatomical impairment (Bruno *et al.*, 2012).

Face recognition is a critical cognitive function for daily social interactions, and it probably played an essential role in the evolution of mankind (Kanwisher and Yovel, 2006). In the past two decades, functional neuroimaging studies have identified the main regions in the occipital-temporal cortex that are involved in face recognition: namely, the occipital face area in the occipital cortex, the fusiform face area in the fusiform gyrus, and the superior temporal sulcus face area (Kanwisher *et al.*, 1997; Haxby *et al.*, 2000; Hoffman and Haxby, 2000). Other patterns of brain activation in the right frontal and parietal lobes have been specifically reported during self-face recognition, and these patterns contrast with those observed during the perception of other people’s (both familiar and unfamiliar) faces (Devue and Brédart, 2011). This specificity of neuronal activation during self-face recognition probably affects interactions with the autonomic brain regions, generating an SSR with an increased area in both minimally and fully conscious subjects when one’s own face is shown. However, in MCS patients, this difference was subtle and not statistically significant when the condition “self-face” was compared to the conditions “unknown” and “well-known,” demonstrating similar sympathetic nervous system activation. This finding may reflect the loss of specificity in the interaction between face recognition and autonomic brain areas in some patients in an MCS, probably associated with a difficulty of differentiating one’s own face from those of others.

The only patient with UWS who showed a greater SSR evoked by self-face presentation (i.e. patient no. 6 in Tables 1 and 2) had a good recovery of consciousness in the subsequent weeks. He was diagnosed as being in an MCS (with a Coma Recovery Scale Revised score of 17) in a new evaluation performed 4 weeks after the study. This result is consistent with previous reports suggesting that the presence of cortical face processing may be associated with the emergence from UWS (Menon *et al.*, 1998; Sharon *et al.* 2013). However, these data should be interpreted with extreme caution, because they may be the effect of a random variation of the SSR area among the conditions, rather than a sign of covert consciousness. On the contrary, the percentage of MCS patients who showed a greater SSR area evoked by the self-face compared with other conditions was higher than would be expected from random variations. Finally, one healthy subject did not have a greater SSR area during self-face presentation. This result suggests that the interaction between the neural functions involved in self-face recognition and sympathetic control may have a physiological degree of variation among normal subjects.

This study has some limitations. Patients with severe disorders of consciousness frequently suffer from neuropathies, as a consequence of intensive care treatments and complications (Bagnato *et al.*, 2011). Although both the sensory nerve conduction of the ulnar nerve and the SSR response evoked by an electrical shock were preliminarily evaluated, we cannot completely exclude the possibility that peripheral autonomic neuropathy may have affected the SSRs in some patients. Moreover, UWS patients do not exhibit visual fixation, and some MCS patients show an inconstant fixation only in response to visual stimuli. To overcome this difficulty, we used a video projector that allowed the reproduction of large images. Nevertheless, we cannot be absolutely sure that the visual stimuli were always presented properly in the patient’s visual field. Eventually, patients in an MCS may display a highly fluctuating level of consciousness. Although we distributed the visual stimuli in four blocks over two different days, a varying level of consciousness during the experiment may have affected the results in some patients. Nevertheless, this is a common limitation of all techniques that require a constant level of arousal, such as functional neuroimaging and electroencephalographic studies. Finally, we did not evaluate other measures of autonomic nervous system activation, such as heart rate changes.

## Conclusions

These results show that the evaluation of involuntary responses mediated by the autonomic nervous system may help to differentiate conscious from unconscious subjects. This finding is particularly relevant in light of the unacceptably high rate of misdiagnosis of UWS versus MCS, when the evaluation is based on clinical assessment alone (Schnakers *et al.*, 2009). Functional neuroimaging and advanced electroencephalographic studies may help to differentiate conscious from unconscious patients, but these studies are expensive and/or require advanced skills (Harrison and Connolly, 2013). Previous preliminary research suggested that autonomic changes with possible emotional value can be induced by complex stimuli in patients with disorders of consciousness (Riganello *et al.*, 2010; Machado *et al.*, 2011). These studies evaluated heart rate changes in response to different auditory stimuli, but were not specifically designed for evaluating the changes related to a possible self-awareness occurrence.

More recently, responses to visual stimuli in patients with disorders of consciousness have been evaluated in eye-tracking studies (Trojano *et al.*, 2012). This methodology seems to be a promising approach for differentiating patients with different levels of consciousness. Moreover, it has been reported that the visual pursuit induced by moving a mirror in front of patients with severe disorders of consciousness is associated with the sympathetic/ parasympathetic balance and the autonomic functional state (Riganello *et al.*, 2013). These results are in line with our findings. Taken together, these previous results and those reported here suggest that the response of the autonomic system to visual stimuli deserves to be further explored in future studies (e.g. by combining eye tracking with SSR evaluation).

The SSR evoked by visual stimuli, including one’s own face, can be obtained via a simple and inexpensive test, and this approach has potential diagnostic implications in patients with disorders of consciousness. The technique may be particularly applied in patients with a very low level of responsiveness, in whom the recoding of autonomic responses may offer evidence of a subtending consciousness. This utilization is very important, because the evaluation of purposeful behaviors by viewing the self-face through a mirror is part of the Coma Recovery Scale, which is considered the gold standard for clinical assessment of patients with disorders of consciousness (Seel *et al.*, 2010). Although future studies in larger numbers of patients are needed, we suggest that the SSR evoked by visual stimuli, including self-face presentation, may be a simple and useful test to increase diagnostic accuracy during the assessment of patients with severe disorders of consciousness.

## Supplementary Material

Supplementary Data
