# Supplementary material for: Autonomic correlates of seeing one’s own face in patients with disorders of consciousness
Source: Neurosci Conscious. 2015 Jul 30;2015(1):niv005. doi: 10.1093/nc/niv005 (PMC6307552; doi:10.1093/nc/niv005)
Supplement: Supplementary Data [file Supplemental_Matherial_Post-hoc_analyses.pdf › Supplemental_Matherial_Post-hoc_analyses.pdf]

Tukey HSD test
Approximate Probabilities for Post Hoc Tests. In red are p values < 0.05.

Cell No.	Group	CONDITIO	{1} 659,17	{2} 566,24	{3} 749,61	{4} 559,95	{5} 731,13	{6} 763,57	{7} 1486,0	{8} 272,20	{9} 269,06	{10} 261,06	{11} 147,22	{12} 648,11	{13} 367,13	{14} 1120,4	{15} 51,232	{16} 30,357	{17} 111,38	{18} 113,11	{19} 142,86	{20} 181,95	{21} 76,411
1	Controls	Board		1,000000	1,000000	1,000000	1,000000	1,000000	0,000115	1,000000	0,999449	0,999272	0,984132	1,000000	0,999993	0,995131	0,999995	0,926566	0,979790	0,980441	0,989276	0,995703	0,962719
2	Controls	Snake	1,000000		0,999943	1,000000	0,999990	0,999823	0,000048	0,999992	1,000000	0,999986	0,998556	1,000000	1,000000	0,964719	0,989574	0,999999	0,997636	0,997747	0,999084	0,999762	0,994131
3	Controls	Stylized face	1,000000	0,999943		0,999904	1,000000	1,000000	0,001174	0,992689	0,992118	1,000000	0,925577	1,000000	0,999581	0,999730	0,837085	0,802141	0,999990	0,918800	0,946039	0,971055	0,874430
4	Controls	Scream	1,000000	1,000000	0,999904		0,999981	0,999717	0,000048	0,999995	0,999993	0,999990	1,000000	1,000000	1,000000	0,960746	0,990917	0,985848	0,998019	1,000000	0,999251	0,999812	0,994971
5	Controls	Unknown	1,000000	0,999990	1,000000	0,999981		1,000000	0,000706	0,995413	0,995024	0,993892	0,943021	1,000000	0,999793	0,999465	0,865034	0,833256	0,935073	0,936616	0,999997	0,979261	0,898293
6	Controls	Known	1,000000	0,999823	1,000000	0,999717	1,000000		0,001718	0,989860	0,989112	0,987014	0,910245	1,000000	1,000000	0,999845	0,814097	0,776946	0,901310	0,903349	0,934205	0,999998	0,854403
7	Controls	Face	0,000115	0,000048	0,001174	0,000048	0,000706	0,001718		0,031995	0,031090	0,028886	0,009658	0,475282	0,073345	1,000000	0,006906	0,005638	0,012246	0,012447	0,016432	0,023477	0,884901
8	MCS	Board	1,000000	0,999992	0,992689	0,999995	0,995413	0,989860	0,031995		1,000000	1,000000	1,000000	0,985255	1,000000	0,022219	1,000000	1,000000	1,000000	1,000000	1,000000	1,000000	1,000000
9	MCS	Snake	0,999449	1,000000	0,992118	0,999993	0,995024	0,989112	0,031090	1,000000		1,000000	1,000000	0,983797	1,000000	0,021108	1,000000	1,000000	1,000000	1,000000	1,000000	1,000000	1,000000
10	MCS	Stylized face	0,999272	0,999986	1,000000	0,999990	0,993892	0,987014	0,028886	1,000000	1,000000		1,000000	0,979557	1,000000	0,018501	1,000000	1,000000	1,000000	1,000000	1,000000	1,000000	1,000000
11	MCS	Scream	0,984132	0,998556	0,925577	1,000000	0,943021	0,910245	0,009658	1,000000	1,000000	1,000000		0,792964	0,999993	0,002355	1,000000	1,000000	1,000000	1,000000	1,000000	1,000000	1,000000
12	MCS	Unknown	1,000000	1,000000	1,000000	1,000000	1,000000	1,000000	0,475282	0,985255	0,983797	0,979557	0,792964		0,999683	0,865695	0,986328	0,980333	0,995969	0,996130	1,000000	0,999361	0,991521
13	MCS	Known	0,999993	1,000000	0,999581	1,000000	0,999793	1,000000	0,073345	1,000000	1,000000	1,000000	0,999993	0,999683		0,090939	0,999998	0,999995	1,000000	1,000000	1,000000	1,000000	1,000000
14	MCS	Face	0,995131	0,964719	0,999730	0,960746	0,999465	0,999845	1,000000	0,022219	0,021108	0,018501	0,002355	0,865695	0,090939		0,336077	0,303210	0,440710	0,443906	0,500023	0,575612	0,998111
15	UWS	Board	0,999995	0,989574	0,837085	0,990917	0,865034	0,814097	0,006906	1,000000	1,000000	1,000000	1,000000	0,986328	0,999998	0,336077		1,000000	1,000000	1,000000	1,000000	1,000000	1,000000
16	UWS	Snake	0,926566	0,999999	0,802141	0,985848	0,833256	0,776946	0,005638	1,000000	1,000000	1,000000	1,000000	0,980333	0,999995	0,303210	1,000000		1,000000	1,000000	1,000000	1,000000	1,000000
17	UWS	Stylized face	0,979790	0,997636	0,999990	0,998019	0,935073	0,901310	0,012246	1,000000	1,000000	1,000000	1,000000	0,995969	1,000000	0,440710	1,000000	1,000000		1,000000	1,000000	1,000000	1,000000
18	UWS	Scream	0,980441	0,997747	0,918800	1,000000	0,936616	0,903349	0,012447	1,000000	1,000000	1,000000	1,000000	0,996130	1,000000	0,443906	1,000000	1,000000	1,000000		1,000000	1,000000	1,000000
19	UWS	Unknown	0,989276	0,999084	0,946039	0,999251	0,999997	0,934205	0,016432	1,000000	1,000000	1,000000	1,000000	1,000000	1,000000	0,500023	1,000000	1,000000	1,000000	1,000000		1,000000	1,000000
20	UWS	Known	0,995703	0,999762	0,971055	0,999812	0,979261	0,999998	0,023477	1,000000	1,000000	1,000000	1,000000	0,999361	1,000000	0,575612	1,000000	1,000000	1,000000	1,000000	1,000000		1,000000
21	UWS	Face	0,962719	0,994131	0,874430	0,994971	0,898293	0,854403	0,884901	1,000000	1,000000	1,000000	1,000000	0,991521	1,000000	0,998111	1,000000	1,000000	1,000000	1,000000	1,000000	1,000000	
